# Molecular Epidemiology of Endemic Human T-Lymphotropic Virus Type 1 in a Rural Community in Guinea-Bissau

**DOI:** 10.1371/journal.pntd.0001690

**Published:** 2012-06-12

**Authors:** Carla van Tienen, Thushan I. de Silva, Luiz Carlos Junior Alcantara, Clayton O. Onyango, Sheikh Jarju, Nato Gonçalves, Tim Vincent, Peter Aaby, Hilton Whittle, Maarten Schim van der Loeff, Matthew Cotten

**Affiliations:** 1 Virology, Medical Research Council, Fajara, The Gambia; 2 Medical Microbiology & Infectious Diseases, Erasmus Medical Centre, Rotterdam, The Netherlands; 3 UCL Centre for Medical Molecular Virology, Division of Infection and Immunity, University College London, London, United Kingdom; 4 Public Health Advanced Laboratory, Gonçalo Moniz Research Center, Oswaldo Cruz Foundation, Salvador, Bahia, Brazil; 5 Bahia School of Medicine and Public Health Foundation for Science Development, Salvador, Bahia, Brazil; 6 KEMRI-Wellcome Trust Programme, Kilifi, Kenya; 7 Projecto de Saúde de Bandim, Bissau, Guinea-Bissau; 8 Projecto de Saúde de Bandim, Indepth Network, Bissau, Guinea-Bissau; 9 Health Service of Amsterdam (GGD) and Academic Medical Centre, Amsterdam, The Netherlands; 10 Wellcome Trust Genome Campus, Wellcome Trust Sanger Institute, Hinxton, Cambridge, United Kingdom; George Mason University, United States of America

## Abstract

**Background:**

Human T-Lymphotropic Virus Type 1 (HTLV-1) infection causes lethal adult T-cell leukemia (ATL) and severely debilitating HTLV-associated myelopathy/tropical spastic paraparesis (HAM/TSP) in up to 5% of infected adults. HTLV-1 is endemic in parts of Africa and the highest prevalence in West Africa (5%) has been reported in Caio, a rural area in the North-West of Guinea-Bissau. It is not known which HTLV-1 variants are present in this community. Sequence data can provide insights in the molecular epidemiology and help to understand the origin and spread of HTLV-1.

**Objective:**

To gain insight into the molecular diversity of HTLV-1 in West Africa.

**Methods:**

HTLV-1 infected individuals were identified in community surveys between 1990–2007. The complete Long Terminal Repeat (LTR) and p24 coding region of HTLV-1 was sequenced from infected subjects. Socio-demographic data were obtained from community census and from interviews performed by fieldworkers. Phylogenetic analyses were performed to characterize the relationship between the Caio HTLV-1 and HTLV-1 from other parts of the world.

**Results:**

LTR and p24 sequences were obtained from 72 individuals (36 LTR, 24 p24 only and 12 both). Consistent with the low evolutionary change of HTLV-1, many of the sequences from unrelated individuals showed 100% nucleotide identity. Most (45 of 46) of the LTR sequences clustered with the Cosmopolitan HTLV-1 subtype 1a, subgroup D (1aD). LTR and p24 sequences from two subjects were divergent and formed a significant cluster with HTLV-1 subtype 1g, and with the most divergent African Simian T-cell Lymphotropic Virus, Tan90.

**Conclusions:**

The Cosmopolitan HTLV-1 1aD predominates in this rural West African community. However, HTLV-1 subtype 1g is also present. This subtype has not been described before in West Africa and may be more widespread than previously thought. These data are in line with the hypothesis that multiple monkey-to-man zoonotic events are contributing to HTLV-1 diversity.

## Author Summary

Human T-Lymphotropic Virus type 1 (HTLV-1) affects millions of people worldwide. It is very similar to Simian T-Lymphotropic Virus, a virus that circulates in monkeys. HTLV-1 causes a lethal form of leukemia (Adult T-cell Leukemia) and a debilitating neurological syndrome (HTLV-associated myelopathy/tropical spastic paraparesis) in approximately 5% of infected people. Based on sequence variation, HTLV-1 can be divided into 7 subtypes (1a–1g) with the Cosmopolitan subtype 1a further subdivided into subgroups (A–E). We examined HTLV-1 diversity in a rural area in Guinea-Bissau, a country in West Africa with a high HTLV-1 prevalence (5%). We found that most viruses belong to the Cosmopolitan subtype 1a, subgroup D, but 2 viruses belonged to subtype 1g. This subtype had thus far only been found in monkey hunters in Cameroon, who were probably recently infected by monkeys. Our findings indicate that this subtype has spread beyond Central Africa. An important, unresolved question is whether persons with this subtype were infected by monkeys or through human-to-human transmission.

## Introduction

The retrovirus Human T-Lymphotropic Virus type 1 (HTLV-1) was first discovered in 1979 [1]. Approximately 5% of HTLV-1 carriers develop Adult T-cell Leukemia (ATL) or HTLV-associated myelopathy/tropical spastic paraparesis (HAM/TSP). ATL (and other HTLV-1 associated diseases have been described in West African individuals [2], [3], [4]. It is not known how many people are infected worldwide [5], but the prevalence is high in Japan, Africa, the Caribbean and South America (reviewed in [6]). Transmission of HTLV-1 is vertical (mainly through prolonged breastfeeding), sexual and via contaminated blood products (reviewed in [6]). HTLV-1 infection is believed to have originated in multiple zoonotic events from monkeys. Cross-species transmission of HTLV-1 from monkey to men is probably ongoing and still actively contributing to the endemic [7], [8].

HTLV-1 is an ancient retrovirus with a very low evolutionary rate (especially compared to HIV) [9]. The Simian T-cell Lymphotropic Virus and HTLV are together referred to as Primate T-cell Lymphotropic Virus (PTLV). Currently, 7 different HTLV-1 subtypes, based primarily on the nucleotide sequence of the LTR region, have been described; the Cosmopolitan subtype (1a) that has spread worldwide and is further divided into subgroups (A–E), 4 African subtypes (1b, 1d, 1e, 1f) and a Melanesian/Australian subtype (1c). In 2005 a new African subtype g was described by Wolfe et al. based on sequence data from two infected subjects [7]. The origins of HTLV-1 are believed to be either in Asia or Africa and current data do not provide clear support for one or the other continent. The most divergent STLV-1 strain thus far identified is Asian (MarB43) suggesting that MarB43 separated earlier from an ancestral virus than the PTLV-1s found in Africa [10] and supporting a hypothesis that the origin of PTLV-1 lies in Asia [10]. On the other hand, only HTLV-1 subtype a has been found in Asia, while all known HTLV-1 subtypes are present in Africa, providing support for an African origin of HTLV-1 [11]. Additional samples and phylogenetic data are needed to answer this question [12].

After an initial dispersion in Asia and Africa, the Cosmopolitan subtype 1a spread worldwide and is thought to have been introduced into the Americas during the post-Columbian slave trade [13]. In people of African descent in South America, mainly Cosmopolitan subtype 1a subgroup A is found [13]. In Africa, subgroup A has been found primarily in Southern Africa while in West Africa (the origin of a large proportion of the slaves that were taken to South America) only subgroups C and D have been identified so far [14]. This presents a puzzle - subgroup A is common in South Americans of African decent yet uncommon (at least based on the currently available sequence data) in the African regions from which a large proportion of slaves were brought to South America. Additional HTLV-1 sequence data from West Africa are needed to resolve this enigma.

The study area of the current analysis is a rural community in Guinea-Bissau and has the highest reported HTLV-1 prevalence in West Africa (5% in 2007) [15], but no sequence data exist from this area and the subtypes of HTLV-1 circulating in the area are not yet identified. Also, the contribution of the various HTLV transmission routes in this community is not well documented . To examine the molecular epidemiology and to define the routes of transmission of HTLV-1 in the Caio community in Guinea Bissau, we characterized the HTLV-1 LTR and p24 region from a collection of DNA samples isolated from children and adults from this cohort. We describe highly conserved examples of HTLV-1 subtype d, most closely related to isolates reported from Bissau and two examples of subtype g, which is the first time this subtype has been reported in West Africa.

## Methods

### Ethics statement

Informed consent was obtained from all the study participants. From 2003 onwards, written informed consent was obtained as required by the Gambian Ethics Committee. In studies prior to the 2003 study, written informed consent was not required by the Gambia Government/MRC MRC Laboratories Joint Ethics Committee nor by the Ministry of Health of Guinea-Bissau and thus verbal consent was obtained. The documentation of the informed consent was done in a two-step process. After information on the study was given by the field worker, and after having responded to any questions that the prospective participant might have, the field worker asked whether the participant was willing to participate in the study. The field worker noted down the answer by ticking one of two boxes (Accepting to participate in study or not); a separate question was asked about providing a blood sample and the answer to this question was noted separately.. All studies, (both prior to and after 2003), were approved by the Gambia Government/MRC MRC Laboratories Joint Ethics Committee and by the Ministry of Health of Guinea-Bissau.

### Study population and samples

The study area, Caio, is a settlement of 10 small villages with 10,000 inhabitants in North-Western Guinea-Bissau and has been described in detail [16]. A rolling census has been performed in the area since 1988 as part of a research project on HIV-2 [17]. In 1990, 1997 and 2007, population surveys were conducted to study the prevalence, incidence and risk factors of HIV-1, HIV-2, and HTLV-1 infection and these surveys included approximately 75% of the adult population [18]. A cohort was established to study the natural history of HIV-2; included were all HIV-2 infected adults and a similar number of controls, matched for age, sex, and village. This cohort was established in 1991, and subjects were examined in 1996, 2003, and 2006. In addition, in 2004 a cross-sectional study was performed to examine mother-to-child transmission of HTLV-1 [19]. HTLV-1 prevalence has been stable at around 5% in Caio throughout this period [15].

In the studies conducted in 2003, 2006 and 2007, DNA was extracted from blood samples (whole blood and peripheral blood mononuclear cells), and stored for molecular analyses. Because these samples have been used in different studies, not each HTLV-1 infected individual had a stored sample; some samples had been depleted during other studies or could not be traced or storage had failed (e.g. freezer breakdowns). The available samples were used for HTLV-1 sequencing for the current study.

Samples for LTR sequencing were selected in 2010 from stored DNA samples. All complete LTR sequences obtained from mother-child pairs from the vertical transmission study in 2004 were also used in the current analysis (accession numbers JN655856 to JN655872). [19]. Samples for p24 sequencing were randomly selected in 2009 from stored DNA samples for an as yet unpublished immunological study.

### HTLV-1 diagnosis

In the 1997, 2003 and 2004 studies, plasma samples were tested for the presence of antibodies to HTLV-1 and HTLV-2 using a Murex HTLV-1+2 ELISA (Abbott Murex Diagnostics, Dartford, UK). Reactive samples were retested by the same assay. HTLV-1 infection status was further determined by PCR using primers targeted to the tax/rex gene [20]. Each PCR reaction also included 10 copies of phage lambda DNA. This was co-amplified with specific primers in the same reaction to control for non-specific inhibition. Because the tax/rex primers amplified both HTLV-1 and HTLV-2 sequences, the amplicons were digested with the restriction enzyme *Sau* 3A (which cuts only HTLV-1) to distinguish products from the two viruses. In this study all PCR-positive samples were confirmed to be HTLV-1 by *Sau* 3A digestion. The HTLV-1 testing in the 2007 study (2 ELISAs and PCR confirmation) and the HIV testing of previous studies have been described in detail [15].

### HTLV-1 LTR and p24 PCR and sequencing

Amplification of the HTLV-1 proviral DNA was performed with nested PCR. PCR primers were optimized for a melting temperature (Tm) between 57 and 60°C and are listed in Table 1. The complete LTR region was obtained by amplifying two overlapping fragments similar to the strategy used by Salemi et al. [21]. For each patient sample, a 438 bp 5′ LTR-gag (primers MO195-198) and a 475 bp tax-3′LTR fragment (primers MO199-202) were obtained. The p24 coding region was amplified on an 840 bp fragment using the nested primers MO076-079.

**Table 1:** Primers used for HTLV-1 LTR and p24 PCR and sequencing.

Primer	Function	Sequence (5′ to 3′)	Comment
MO195	5′LTR-*gag* OF	AACTAGCAGGAGTCTATAAAAGCG	based on AV117
MO196	5′LTR-*gag* OR	AAAGATTTGGCCCATTGCCTAG	based on AV118
MO197	5′LTR-*gag* IF	ACAGTTCAGGAGGGGGCTC	based on AV119
MO198	5′LTR-*gag* IR	TAGGGAATAAAGGGGCGCTC	based on AV120
MO199	Tax-3′ LTR OF	ACTCACACGGCCTCATACAG	based on AV121
MO200	Tax-3′ LTR OR	ACGCAGTTCAGGAGGCAC	based on AV122
MO201	Tax-3′ LTR IF	CTGTTTGAAGAATACACCAACATCC	based on AV123
MO202	Tax-3′ LTR IR	CTCAACCGGCGTGGATGG	based on AV124
MO076	p24 OF	TCCCTCCTAGCCAGCCTAC	
MO079	p24 OR	TCTCGCTTCCAGTGAGTTGG	
MO077	p24 IF	CATCCAAACCCAAGCCCAGA	
MO078	p24 IR	CTCCAGTGGCCTGCTTTCC	

OF, outer forward; OR, outer reverse; IF, inner forward; IR, inner reverse.

Primers MO195-MO202 based on, but not identical to primers described in Salemi et al. [21].

PCR products were purified using Qiaquick Gel Extraction (Qiagen) and sequenced using the inner PCR primers from both directions by Macrogen (www.macrogen.com). Complete LTR and p24 sequences were assembled and ambiguities were resolved using the sequence alignment editor BioEdit. [22] All new sequences generated here have been deposited in Genbank with the following accession numbers (JQ583778 to JQ583845) (Table S1).

### Phylogenetic analysis

Multiple sequence alignments of the LTR and the related sequences in the GenBank/EMBL database were performed with the Clustal [23] and Dambe softwares [24]. A minimal editing of the alignment was performed manually with GeneDoc [25]. Neighbor-joining (NJ) and maximum-likelihood (ML) phylogenetic analyses were performed with PAUP*, version 4.0b10 [26]. The Tamura-Nei evolutionary model (taking into account a different substitution rate for transversions, purine and pyrimidine transitions and allowing an intersite substitution rate heterogeneity modelled with a gamma distribution) was chosen as best model (alpha parameter of 0.6) using Modeltest 3.06 [27]. The NJ tree was constructed with an optimized nucleotide substitution rate matrix and gamma shape parameter using empirical base frequencies. The reliability of the NJ trees was evaluated by analyzing 1,000 bootstrap replicates and bootstrap values of >60% were considered significant. For the ML tree reconstruction a heuristic search with the subtree-pruning-regrafting branch swapping algorithm was performed using the NJ tree as starting tree including its optimised parameters. A likelihood ratio test was used to calculate the statistical support for the branches (expressed in p-values). Trees were drawn with TreeView 1.4 [28].

## Results

### Study subjects and sequences

HTLV-1 LTR and/or p24 sequences were obtained from 72 individuals (Table 2). In order to obtain LTR sequences from persons who had been infected relatively recently (between 1997 and 2007) or earlier (prior to 1990 or 1997), samples were selected from individuals diagnosed with HTLV-1 in 2007. Other samples were randomly selected from the 2003 study. Of the 72 individuals yielding sequence, 14 (19%) were diagnosed with HTLV-1 in 1990, 17 (24%) in 1997, 5 (7%) in 2003 and 28 (40%) in 2007 (Table S1). Twenty-five (35%) individuals were co-infected with HIV; 1 was HIV-1, 19 were HIV-2 and 5 were HIV-1/HIV-2 dual co-infected. Eight (11%) sequences came from children, who were all diagnosed with HTLV-1 in 2004 and who were all HIV negative.

**Table 2:** Characteristics of subjects of whom HTLV-1 LTR and/or p24 sequences were obtained from Caió, Guinea-Bissau, 2003–2007.

Characteristic	
Total number of subjects of whom sequences were available	72
Number of women (%)	54 (75)
Number of children (aged <15 years) (%)	8 (11)
Median age at diagnosis in years (IQR)	41 (24–64)
Number of subjects co-infected with HIV (%)	25 (35)
Subjects of whom LTR sequence was available(%)	48 (67)
Subjects of whom p24 sequence was available (%)	36 (50)
Subjects of whom both LTR and p24 sequence were available (%)	12 (17)

IQR, interquartile range.

Information on relationships between individuals from whom sequences were available was obtained from census data and interviews; there was one married couple (Caio5846 & Caio5884), 2 siblings (Caio4757 & Caio4758) and 6 mother-child pairs (Caio4634 & Caio4635; Caio4647 & Caio4650; Caio4658 & Caio4659; Caio4743 & Caio4745; Caio4671& Caio4702; Caio4799 & Caio4801). Within each of these 8 family pairs, the viruses showed 100% identical sequences.

### Molecular typing

**Figure 1:**
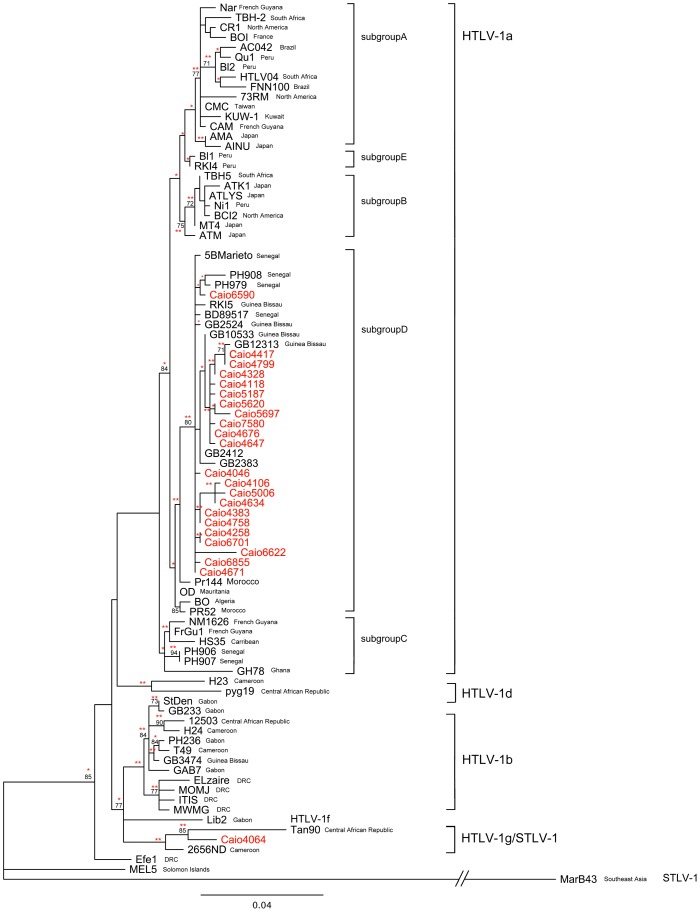
ML tree of 630-bp LTR sequences from 48 Caio infected subjects (Caio sequences in red). Also included are 66 STLV/HTLV-1 reference sequences (in black); Mel5 was used as an outgroup. The names of the countries of origin are denoted in brackets (CAR, Central African Republic). The phylogenetic tree was inferred under a Tamura-Nei evolutionary model with a gamma model of among-site substitution rate heterogeneity. Bootstrap values using 1000 replicates of ≥60 are indicated on the branches. The asterisks indicate significant branch length on ML testing (* p-value≤0.001; ** p-value≤0.05). The scale bar represents the number of nucleotide substitutions per site. The scale of the MarB43 is not on scale because it did not fit in the figure. Sequences identical to Caio4676 are Caio4126, Caio4142, Caio4315, Caio4768, Caio5324, Caio5801, Caio6460, Caio6936 and Caio7120. Sequence identical to Caio4647 is Caio4650. Sequence identical to Caio4799 is Caio4801. Sequences identical to Caio5620 are Caio4743, Caio4745 and Caio5883. Sequences identical to Caio5187 are Caio5846, Caio5884 and Caio5931. Sequences identical to Caio4634 are Caio4635 and Caio5448. Sequences identical to Caio4758 are: Caio4658, Caio4659 and Caio4757. Sequence identical to Caio4671 is Caio4702. Sequences identical to 7580 are 7421 and 7661. Only one example of each set of identical sequences was included in the analysis.

First, LTR sequences were analyzed to identify the HTLV-1 subtypes present in the village (Table S1). Identical HTLV-1 LTR sequences were found in known relatives but were also obtained from 10 unrelated individuals with no known epidemiological links (not related or known sexual partners) (Figure 1). Stringent control measures were in place to prevent cross-contamination during sample handling and PCR, and there was a consistent absence of product in all negative controls (run with each set of PCR reactions). In addition there was no clustering of identical sequences by day of sample preparation. The nonidentical sequences exhibited 1 to 3% divergence for the LTR.

A phylogenetic tree was constructed with all nonidentical LTR sequences (Figure 1 - the identical sequences are listed in the figure legend). With a single exception the Caio sequences (marked in red) clustered with HTLV-1 subgroup D reference strains isolated from pregnant women from Bissau (capital of Guinea-Bissau) and other West and North African individuals.

One virus LTR sequence, Caio4064, was very divergent (6%) from the major group of Caio subtype 1a isolates. Caio4064 clustered significantly with an STLV-1 sequence obtained from a wild African green monkey in the Central African Republic [29] (Tan90) and with HTLV-1 subtype 1g isolated from a Cameroonian monkey hunter (2656ND) [7]. The Caio4064 sample was obtained from a 65-year old woman diagnosed with HTLV-1 (proviral load: 22 copies per 10^5^ cells) and HIV-2 (viral load: undetectable) infection in 1990. She had not travelled outside of Guinea-Bissau and had not been bitten by a monkey or hunted monkeys (information obtained in the 1997 survey). Her husband was HTLV-1 negative in the most recent survey (2007).

**Figure 2:**
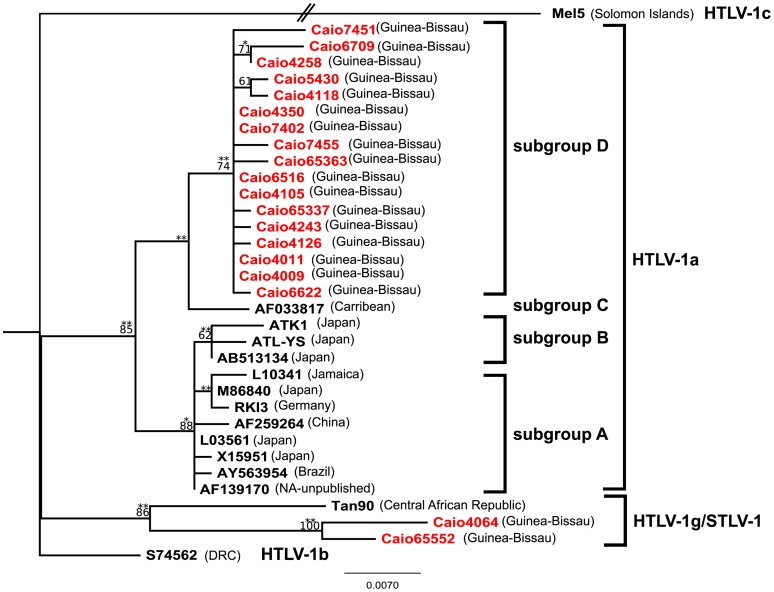
ML tree of 647-bp p24 sequences from 36 Caio infected subjects (Caio sequences in red). Also included are 15 STLV/HTLV-1 reference sequences (in black); Mel5 was used as an outgroup. The countries of origin are denoted in brackets (CAR, Central African Republic; NA, not available). The phylogenetic tree was inferred under a Tamura-Nei evolutionary model with a gamma model of among-site substitution rate heterogeneity. Bootstrap values using 1000 replicates of ≥60 are indicated on the branches. The asterisks indicate significant branch length on ML testing (* p-value≤0.001; ** p-value≤0.05). The scale bar represents the number of nucleotide substitutions per site. Sequences identical to Caio4009 are Caio4072, Caio4106, Caio4112, Caio4211, Caio4315, Caio4328, Caio4358, Caio4383, Caio4417, Caio4418, Caio5801, Caio6473, Caio6590, Caio65396 and Caio65407. Sequence identical to Caio65363 is Caio65571. Sequence identical to Caio4258 is Caio65325. Only one example of each set of identical sequences was included in the analysis.

Further evidence for the presence of subtype 1g was obtained by examining a set of p24 sequences obtained from 36 individuals (Table S1, Figure 2 - the identical sequences are listed in the figure legend). Although both LTR and p24 sequences were not available for all subjects, there was sufficient overlap between the two sets of sequences to make some important conclusions. Sixteen individuals with no epidemiological links to each other (not related and not sexual partners) had identical p24 sequences. A ML-tree was constructed from the non-identical sequences; 32 of the Caio p24 sequences formed a single cluster. Consistent with the LTR phylogeny, Caio4064 p24 clustered significantly with the Tan90 p24 and an additional Caio sample, Caio65552. Caio65552 was obtained from a 62 year old man who reported having hunted monkeys in the Caio area, but had no recollection of being bitten by a monkey. The subject was diagnosed with HTLV-1 (proviral load: 2199 per 10^5^ cells) and HIV-2 (viral load: undetectable) in 1997. He was not married and had no epidemiological link with the patient providing Caio4064. No samples were available from the mothers of subjects providing Caio4064 or Caio65552 to examine vertical transmission.

## Discussion

This is the first characterization of the sequence diversity of HTLV-1 within a single, endemic community in West Africa with sequences obtained from 72 adults and children. An important feature of this study is that individuals were screened for HTLV-1 in population surveys and were not selected from a clinical setting, thus avoiding selection bias towards diseased individuals. Most of the sequences identified belonged to HTLV-1 Cosmopolitan subtype 1a subgroup D. However, two subjects were found to be infected with a divergent strain of HTLV-1 which clustered significantly with subtype 1g. This is the first time that this novel subtype has been isolated from individuals in West Africa and suggests this subtype has spread into the human population outside of Cameroon, either by human-to-human or interspecies transmission.

Remarkable genetic stability in 1aD sequences was observed and in particular, 10 identical LTR sequences and 16 identical p24 sequences were found in people with no identified epidemiological links. These infections differed in time of diagnosis, route of infection (vertically and horizontally) and the length of the infection (a few years vs. >14 years). This observation is consistent with the very low evolutionary rate of HTLV-1 (estimated at 7.06×10^−7^−1.38×10^−5^ substitutions per site per year) [9], which is probably explained by clonal expansion of the virus (rather than viral replication) [30]. Whether this genetic stability is also influenced by host factors and whether specific viral variants are associated with proviral load and/or disease progression are important questions that remain to be elucidated. With such a limited degree of viral variation observed across a community cohort, combined with the low rate of progression to any HTLV-1 associated disease it is difficult to identify such an association. In Caio, 7.1% of HTLV-1 infected individuals were diagnosed with HTLV-associated myelopathy/tropical spastic paraparesis (HAM/TSP) [31], but no data regarding Adult T-cell Leukemia are available. HTLV-1 infection in a large cohort study from Caio was found to be associated with increased mortality, especially among younger people and an increased proviral load was associated with increased mortality [32].

HTLV-1aD was the predominant variant in our study population, consistent with other studies from Bissau and Senegal [33], [34]. Recently, this variant was also described in Europe in patients from West Africa who had emigrated to Portugal [35]. HTLV-1 phylogeny has been examined in a historical context to trace the origin of the virus and movement of people. While slaves who were brought to South America came from West Africa including Guinea-Bissau, surprisingly subgroup D has not been found among African descendants in South America. Subgroup A and C are prevalent among African descendants, for example in Brazil and French Guyana. Subgroup A could have been introduced into Brazil through slaves that came from Southern Africa [14], [36]. Subgroup C has been found in the Noir Marron, a distinct population of African origin, who settled in French Guyana and the majority of whom originated from the Bight of Benin (present-day Togo, Benin and a part of Nigeria) and Gold Coast (a part of Ivory Coast and Ghana) [37]. The fact that subgroup D has not been described in these populations may be due to subgroup C being older and/or a later introduction of subgroup D into West Africa (so that the prevalence was low or absent during the slave trade). It could also be that the number of slaves, originally from the countries where subgroup D was prevalent, was (relatively) low. To answer this question, additional analysis of both host and viral genetic markers is required. More sampling in West Africa and in groups of African descent in South America could shed light on this issue.

The finding of two instances of HTLV-1 subtype 1g, Caio4064 and Caio65552, from the Caio community is of great interest for several reasons. It indicates that this subtype is present beyond Cameroon and can also be found in a ‘normal’ community member, i.e. not only in people with close contact to monkeys such as hunters.

That subtype 1g isolates cluster significantly with Tan90, the most divergent African STLV-1 strain, gives support to an African origin of HTLV-1 with the oldest known STLV-1 as the most recent common ancestor of Caio4064/Caio65552. Although the Asian MarB43 is the most divergent STLV-1, it does not cluster with any known HTLV-1 strains and thus may be only distantly related to STLV subgroups that moved into humans. While most African STLV and HTLV strains cluster together, mainly by geographic location, Asian STLV and HTLV strains only cluster by species, suggesting cross-species transmission has frequently occurred in Africa and not in Asia [11]. No simian strain has been described yet which clusters with HTLV-1a, the most widespread subtype. Whether this is due to relatively infrequent sampling of non-human primates or due to other factors, remains to be resolved [38].

The presence of HTLV-1 subtype 1g infection could be the result of human to human contact (Caio residents do travel extensively [39]). Alternatively, a zoonotic transmission may also explain the presence of this subtype. Hunting of monkeys and butchering for human consumption is commonly practiced [40]. In Caio, captured monkeys are often kept tethered in household compounds for future consumption and HTLV-1 infection has been found to be associated with monkey bites among older persons in the capital Bissau [41]. These social factors, combined with the low evolutionary rate of HTLV-1 [9] are consistent with subtype g virus appearing in Caio due to a transmission from a local monkey. Indeed, the 62 year old Caio man yielding Caio65552 reported hunting monkeys in the area; however, the woman from whom the Caio4064 isolate was obtained, reported that she did not hunt monkeys and was not bitten by one suggesting that her infection came about by another route. Iatrogenic spread should also be considered, as both individuals are older and co-infected with HIV-2 [42], [43].

HTLV-1 infection is considered to be largely an infection of older women [44]. However, 6 young men (16 years of age) were found to have become infected with HTLV-1 between 1997–2007 in the Caio community [15], which led us to consider transmission during traditional local, non-sterile, male circumcision ceremonies. These take place every ten years in a sacred part of the Caio forest. This initiation ritual is exclusively for men and lasts three months. Three men included in the current analysis were known to have participated in the same circumcision ceremony. However, the LTR sequences from the viruses found in these 3 men (yielding Caio5187 & Caio5801 & Caio7580) were not identical and no more closely related to each other than to sequences from the wider Caio population. (i.e. they did not cluster separately from the larger subgroup D cluster (Figure 1)). Both of these features would have been expected if the virus in these three subjects had been transmitted by infected blood in such a ceremony [45], [46]. Thus, although a traditional circumcision ceremony has the potential to spread blood borne viruses, the phylogenetic data clearly do not support the idea that this ceremony was the source of HTLV-1 infection of these young men.

Epidemiological surveillance and further sequencing are recommended to identify (novel) HTLV-1 subtypes [47], [48] and to investigate the spread of subtype 1g. It would be of particular interest to investigate to what extent new introductions of HTLV subtypes (and potentially other retroviruses) [7], [49], [50] occur and whether non-sexual, non-vertical transmission takes place in this community. Further testing of indeterminate HTLV-1/2 ELISA samples found in this community [15] could indicate for example whether HTLV-2 is also present in Caio [51]. This may lead to a better understanding of the spread of other zoonotic events, of which the HIV pandemic is a major example [40], and can have important public health implications.

## Supporting Information

Table S1 **Characteristics of 72 individuals from whom HTLV-1 LTR and/or p24 sequences were obtained, Caió, Guinea-Bissau.**
(DOC)
