# Supplementary material for: Molecular Epidemiology of Endemic Human T-Lymphotropic Virus Type 1 in a Rural Community in Guinea-Bissau
Source: PLoS Negl Trop Dis. 2012 Jun 12;6(6):e1690. doi: 10.1371/journal.pntd.0001690 (PMC3373628; doi:10.1371/journal.pntd.0001690)
Supplement: Table S1 — Characteristics of 72 individuals from whom HTLV-1 LTR and/or p24 sequences were obtained, Caió, Guinea-Bissau. (DOC) [file pntd.0001690.s001.doc]

**Table S1. Characteristics of 72 individuals from whom HTLV-1 LTR and/or p24 sequences were obtained, Caió, Guinea-Bissau.**

| **ID** | **Sex** | **Age at sampling**  **(years)** | **Year of diagnosis** | **Year of sampling** | **Year of last seronegative sample** | **LTR/p24 sequenced** | **Gen Bank Accession number(s)** |
| --- | --- | --- | --- | --- | --- | --- | --- |
| Caio4009 | F | 84 | 1990 | 2003 | NA | p24 | JQ583810 |
| Caio4011 | F | 73 | 1990 | 2003 | NA | p24 | JQ583811 |
| Caio4046 | M | 76 | 1997 | 2003 | NA | LTR | JQ583778 |
| Caio4064 | F | 65 | 1990 | 2003 | NA | LTR + p24 | JQ583779 +  JQ583812 |
| Caio4072 | F | 53 | 1990 | 2003 | NA | p24 | JQ583813 |
| Caio4105 | F | 44 | 1990 | 2003 | NA | p24 | JQ583814 |
| Caio4106 | F | 39 | 1997 | 2003 | 1990 | LTR + p24 | JQ583780  +  JQ583815 |
| Caio4112 | F | 64 | 2003 | 2003 | 1997 | p24 | JQ583816 |
| Caio4118 | F | 69 | 1990 | 2003 | NA | LTR + p24 | JQ583781  + JQ583817 |
| Caio4126 | F | 81 | 1990 | 2003 | NA | LTR + p24 | JQ583795  +  JQ583818 |
| Caio4142 | F | 69 | 1997 | 2003 | NA | LTR | JQ583797 |
| Caio4211 | F | 30 | 2003 | 2003 | NA | p24 | JQ583819 |
| Caio4243 | M | 41 | 1997 | 2003 | 1990 | p24 | JQ583820 |
| Caio4258 | F | 58 | 1990 | 2003 | NA | LTR + p24 | JQ583782  +  JQ583821 |
| Caio4315 | F | 81 | 1990 | 2003 | NA | LTR + p24 | JQ583798  +  JQ583822 |
| Caio4328 | F | 30 | 1990 | 2003 | NA | LTR + p24 | JQ583783  +  JQ583823 |
| Caio4350 | F | 54 | 1990 | 2003 | NA | p24 | JQ583824 |
| Caio4358 | F | 53 | 2003 | 2003 | 1997 | p24 | JQ583825 |
| Caio4383 | F | 49 | 1997 | 2003 | NA | LTR + p24 | JQ583784  +  JQ583826 |
| Caio4417 | F | 78 | 1997 | 2003 | NA | LTR + p24 | JQ583785  +  JQ583827 |
| Caio4418 | F | 48 | 2003 | 2003 | NA | p24 | JQ583828 |
| Caio4634 | F | 33 | 1997 | 2004 | NA | LTR | JN655856 |
| Caio4635 | F | 1 | 2004 | 2004 | NA | LTR | JN655857 |
| Caio4647 | F | 23 | 1997 | 2004 | NA | LTR | JN655858 |
| Caio4650 | F | 3 | 2004 | 2004 | NA | LTR | JN655859 |
| Caio4658 | F | 58 | 1997 | 2004 | 1990 | LTR | JN655860 |
| Caio4659 | F | 1 | 2004 | 2004 | NA | LTR | JN655861 |
| Caio4671 | F | 40 | 1990 | 2004 | NA | LTR | JN655862 |
| Caio4676 | F | 37 | 1997 | 2004 | NA | LTR | JN655863 |
| Caio4702 | F | 1 | 2004 | 2004 | NA | LTR | JN655864 |
| Caio4743 | F | 40 | 1997 | 2004 | 1990 | LTR | JN655865 |
| Caio4745 | M | 7 | 2004 | 2004 | NA | LTR | JN655866 |
| Caio4757 | M | 1 | 2004 | 2004 | NA | LTR | JN655867 |
| Caio4758 | M | 6 | 2004 | 2004 | NA | LTR | JN655868 |
| Caio4768 | F | 40 | 1997 | 2004 | NA | LTR | JN655869 |
| Caio4799 | F | 5 | 2004 | 2004 | NA | LTR | JN655871 |
| Caio4801 | F | 21 | 1997 | 2004 | NA | LTR | JN655872 |
| Caio5006 | F | 64 | 2007 | 2007 | 1997 | LTR | JQ583786 |
| Caio5187 | M | 26 | 2007 | 2007 | 1997 | LTR | JQ583787 |
| Caio5324 | F | 17 | 2007 | 2007 | NA | LTR | JQ583799 |
| Caio5430 | F | 16 | 2007 | 2007 | NA | p24 | JQ583836 |
| Caio5448 | F | 61 | 2007 | 2007 | 1997 | LTR | JQ583796 |
| Caio5620 | F | 57 | 2007 | 2007 | 1990 | LTR | JQ583788 |
| Caio5697 | F | 25 | 2007 | 2007 | 1997 | LTR | JQ583789 |
| Caio5801 | M | 19 | 2007 | 2007 | NA | LTR + p24 | JQ583800  +  JQ583837 |
| Caio5846 | M | 25 | 2007 | 2007 | 1997 | LTR | JQ583805 |
| Caio5883 | M | 18 | 2007 | 2007 | NA | LTR | JQ583804 |
| Caio5884 | F | 23 | 2007 | 2007 | NA | LTR | JQ583806 |
| Caio5931 | M | 25 | 2007 | 2007 | 1997 | LTR | JQ583807 |
| Caio6460 | F | 44 | 2007 | 2007 | 1997 | LTR | JQ583801 |
| Caio6473 | F | 48 | 2007 | 2007 | NA | p24 | JQ583838 |
| Caio6516 | M | 23 | 2007 | 2007 | NA | p24 | JQ583839 |
| Caio65325 | F | 61 | 1990 | 2006 | NA | p24 | JQ583829 |
| Caio65337 | F | 67 | 1997 | 2006 | NA | p24 | JQ583830 |
| Caio65363 | M | 74 | 1997 | 2006 | NA | p24 | JQ583831 |
| Caio65396 | F | 76 | 1990 | 2006 | NA | p24 | JQ583832 |
| Caio65407 | F | 42 | 1997 | 2006 | 1990 | p24 | JQ583833 |
| Caio65552 | M | 58 | 1997 | 2006 | NA | p24 | JQ583834 |
| Caio65571 | F | 63 | 2003 | 2006 | 1997 | p24 | JQ583835 |
| Caio6590 | F | 56 | 2007 | 2007 | 1997 | LTR + p24 | JQ583790  +  JQ583840 |
| Caio6622 | F | 40 | 2007 | 2007 | NA | LTR + p24 | JQ583791  +  JQ583841 |
| Caio6701 | F | 72 | 2007 | 2007 | 1997 | LTR | JQ583792 |
| Caio6709 | M | 18 | 2007 | 2007 | NA | p24 | JQ583842 |
| Caio6855 | F | 23 | 2007 | 2007 | NA | LTR | JQ583793 |
| Caio6936 | F | 26 | 2007 | 2007 | 1997 | LTR | JQ583802 |
| Caio7120 | F | 71 | 2007 | 2007 | NA | LTR | JQ583803 |
| Caio7402 | M | 19 | 2007 | 2007 | NA | p24 | JQ583843 |
| Caio7421 | F | 77 | 2007 | 2007 | 1997 | LTR | JQ583808 |
| Caio7451 | F | 88 | 2007 | 2007 | NA | p24 | JQ583844 |
| Caio7455 | F | 36 | 2007 | 2007 | NA | p24 | JQ583845 |
| Caio7580 | M | 28 | 2007 | 2007 | 1997 | LTR | JQ583794 |
| Caio7661 | M | 28 | 2007 | 2007 | 1997 | LTR | JQ583809 |

F, female; M, male; n, no; y, yes; NA, not available
